# Diagnostic value of neutrophil-to-lymphocyte, platelet-to-lymphocyte, and lymphocyte-to-monocyte ratios for assessing organ and multiorgan involvement in sarcoidosis: a retrospective single-center study

**DOI:** 10.3389/fmed.2026.1767945

**Published:** 2026-03-13

**Authors:** Estefanía Díaz-Martín, Andrea Fernández-Valmaña, Jesús López-Martínez, Alex Mayer-Fuentes, Joan María Mercadé-Torras, María García-González, Laia Mas-Maresma, Blanca Carrillo-Lampe, Joel Font-Majo, Begoña Marí-Alfonso, Carlos Feijoo-Massó

**Affiliations:** Department of Systemic Autoimmune Diseases-Internal Medicine, Parc Taulí Hospital Universitari, Institut d'Investigació i Innovació Parc Taulí (I3PT-CERCA), Universitat Autònoma de Barcelona, Sabadell, Spain

**Keywords:** lymphocyte-to-monocyte ratio, multiorgan involvement, neutrophil-to-lymphocyte ratio, platelet-to-lymphocyte ratio, sarcoidosis

## Abstract

**Background:**

Sarcoidosis is a heterogeneous disease lacking reliable biomarkers for organ involvement. Indices derived from the complete blood count (CBC), including the neutrophil-to-lymphocyte ratio (NLR), platelet-to-lymphocyte ratio (PLR), and lymphocyte-to-monocyte ratio (LMR), have emerged as accessible markers of systemic inflammation.

**Objectives:**

To assess whether CBC parameters and NLR, PLR, and LMR vary across demographic and clinical features in sarcoidosis, and to evaluate their diagnostic performance for organ-specific and multiorgan involvement.

**Methods:**

A retrospective, single-center observational study included adults with sarcoidosis diagnosed between 2000 and 2025. Age- and sex-adjusted logistic regression evaluated associations between hematologic indices at diagnosis and organ involvement. Additional logistic regression using analytical parameters evaluated associations between blood count–derived ratios and multiorgan involvement. Firth’s correction was applied when the events-per-variable ratio was < 10, and analyses were repeated using log-transformed hematologic ratios when skewness coefficient > 2. Diagnostic performance was assessed by receiver operating characteristic (ROC) curve analysis.

**Results:**

A total of 229 patients were included (mean age 51.34 years; 57.64% women). Median values for NLR, PLR, and LMR were 2.57, 158.97, and 3.17, respectively. Higher NLR and PLR were independently associated with extrathoracic lymph node involvement (NLR: OR = 13.42, 95% CI 1.91–94.32, *p* = 0.001, PLR: OR = 1.01, 95% CI 1.00–1.01, *p* = 0.008). NLR was associated with splenic involvement (OR = 83.05, 95% CI 6.75–1021.09, *p* = 0.001); Firth’s correction confirmed the association (OR = 23.72; 95% CI 7.53–74.70). Log-transformed NLR remained associated with splenic (OR = 2.74 per 10% increase, 95% CI 1.92–3.91) and extrathoracic lymph node involvement (OR = 2.17 per 10% increase, 95% CI 1.60–2.94). NLR was associated with multiorgan disease (OR = 24.60, 95% CI 5.90–102.00, *p* < 0.001), and log-transformed NLR showed a consistent association (OR = 2.13 per 10% increase, 95% CI 1.56–2.91). The area under the ROC curve was 0.51 for ≥2 organs, 0.69 for ≥3 organs, and 0.99 for ≥4 organs.

**Conclusion:**

NLR was independently associated with multiorgan disease, splenic and extrathoracic lymph node involvement. PLR was independently associated with extrathoracic lymph node involvement.

## Introduction

Sarcoidosis is a multisystem granulomatous disorder characterized by substantial heterogeneity in clinical expression, influenced by demographic, geographic, and genetic determinants. Onset most often occurs in middle adulthood, with a slight female predominance (1–5). Epidemiologic patterns also demonstrate pronounced global variability, with notably higher prevalence in Northern European populations and disproportionately elevated rates among African Americans (6–10).

Histologically, sarcoidosis is defined by epithelioid non-caseating granulomas, the pathological hallmark of the disease. These granulomas reflect the underlying inflammatory nature of sarcoidosis. They result from an exaggerated innate immune response in which activated macrophages differentiate into epithelioid cells and may fuse into multinucleated giant cells in close interaction with dendritic cells. Antigen-presenting cells subsequently activate CD4^+^ T lymphocytes, promoting a Th1-polarized immune response with increased cytokine production that amplifies macrophage activation and sustains granuloma formation. Regulatory T-cell dysfunction and Th17 responses are also believed to contribute to persistent inflammation and chronic disease. Overall, sarcoidosis represents a dysregulated immune response to unidentified antigens in genetically susceptible individuals, resulting in granulomatous inflammation. Consequently, diagnosis relies on compatible clinical and radiologic findings together with histologic evidence of non-necrotizing granulomas, after exclusion of alternative causes (1–5, 11–13).

Sarcoidosis can affect almost any organ, resulting in marked phenotypic heterogeneity. The clinical spectrum is highly variable, and disease progression is often unpredictable, ranging from incidental, asymptomatic findings to advanced or life-threatening disease. Pulmonary and intrathoracic lymph node involvement are the most frequent manifestations, occurring in over 90% of patients (1–5, 14, 15).

Extrapulmonary disease is common, affecting up to half of patients; however, isolated extrapulmonary involvement is uncommon, occurring in approximately 5–9% of cases (15, 16). Among extrapulmonary sites, the skin is the most frequently affected. Cutaneous sarcoidosis occurs in up to one-third of patients, with erythema nodosum being the most typical presentation. Ocular involvement is also prevalent, most often manifesting as bilateral anterior uveitis. Less commonly affected organs include the joints, liver, spleen, upper airway, kidneys, bone marrow, heart, and nervous system (1–5, 14).

Epidemiologic data reveal a wide spectrum of systemic involvement, ranging from two-organ disease to widespread multisystem presentations. Most studies define “multiorgan” sarcoidosis as involvement of three or more organs, the most commonly used threshold, although some use cut-offs of two or four. Recognition of multiorgan involvement is necessary because the number of organs affected at diagnosis correlates with adverse outcomes (17–19).

Over recent years, considerable effort has been devoted to identifying biomarkers that could improve the diagnosis, monitoring, and risk assessment of patients with sarcoidosis. Despite research, most proposed biomarkers lack sufficient specificity or sensitivity to be used in isolation for clinical decision-making (1, 3). This has led to growing interest in hematologic parameters because they represent a widely accessible, inexpensive, and routinely available source of biological information (20, 21). These include absolute leukocyte counts (neutrophils, lymphocytes, and monocytes), platelet counts, as well as derived indices such as the neutrophil-to-lymphocyte ratio (NLR), platelet-to-lymphocyte ratio (PLR), and lymphocyte-to-monocyte ratio (LMR).

Sarcoidosis patients may present with various cytopenias, most commonly lymphopenia, but also anemia or thrombocytopenia (20), and lymphopenia has been associated with greater disease severity and worse clinical outcomes (22), raising interest in its potential value as a prognostic marker. However, despite these observations, robust and reliable biomarkers for diagnosis, monitoring, and risk stratification remain lacking (23, 24).

Among CBC-derived indices, NLR has been widely proposed as a marker of systemic inflammation across different clinical settings, including malignancy, cardiovascular disease, and critical illness, as reflected in prior studies summarized in the sarcoidosis literature (21, 25). In sarcoidosis, elevated NLR has been associated with pulmonary hypertension (26) and other interstitial lung disease (27–29), and has been proposed as an indicator of systemic inflammation (25, 30).

In contrast, increased PLR and LMR values have been reported in sarcoidosis, but their clinical significance remains uncertain (31). Moreover, data supporting the use of these indices to predict multiorgan involvement, an important determinant of morbidity, are still scarce.

Building on these observations, this study aimed to systematically evaluate whether hematological parameters differ according to demographic factors and patterns of organ involvement in a cohort of patients with sarcoidosis. Specifically, lymphocyte, neutrophil, monocyte, eosinophil, and platelet counts were analyzed, together with NLR, PLR, and LMR, in relation to age, sex, and race/ethnicity, as well as pulmonary involvement (including Scadding stage), extrapulmonary manifestations, and multiorgan involvement.

## Methods

A single-center, retrospective observational study was carried out at Hospital Universitari Parc Taulí (Sabadell, Barcelona, Spain), including all consecutive adults (≥18 years) diagnosed with sarcoidosis between January 2000 and July 2025. The Clinical Research Ethics Committee of Hospital Parc Taulí approved the study. All procedures complied with the Declaration of Helsinki and Good Clinical Practice guidelines. Data were anonymized in accordance with Regulation (EU) 2016/679 and Spanish Organic Law 3/2018 on Data Protection and Digital Rights.

### Study population

The diagnosis of sarcoidosis was established according to the 1999 American Thoracic Society, the European Respiratory Society and the World Association of Sarcoidosis and Other Granulomatous Disorders (WASOG) criteria (12), which require all three of the following: (1) clinical and/or radiological features consistent with sarcoidosis; (2) histologic evidence of non-caseating granulomas; and (3) exclusion of alternative granulomatous diseases. Eligible patients had CBCs performed at diagnosis or within 30 days before diagnosis. Patients with active infection, hematologic malignancy, pregnancy, or exposure to systemic corticosteroids or other immunosuppressants within the four weeks before the CBC were excluded.

### Data collection

Data were collected retrospectively using a standardized data-collection process. All clinical, radiological, histological, and laboratory variables were independently reviewed and validated by two independent reviewers, with discrepancies resolved by consensus. Variables were grouped into the following categories: demographic characteristics, clinical presentation, organ involvement, and analytical parameters.

Ethnicity was categorized following U. S. Food and Drug Administration definitions. For comparative analyses, ethnicity was dichotomized into White versus non-White. Individual non-White subgroups (Black, Hispanic/Latino, and Asian) were described separately to provide detailed demographic characterization.

Clinical and radiological variables included pulmonary, extrapulmonary, and multiorgan involvement, defined as follows:

- Pulmonary involvement was defined and staged using the Scadding scale system on chest radiography or computed tomography: stage I (bilateral hilar lymphadenopathy [BHL] only), stage II (BHL with parenchymal infiltrates), stage III (parenchymal infiltrates without BHL), and stage IV (advanced fibrosis).
- Extrapulmonary involvement was assessed according to the 2014 WASOG criteria (13) across the following organ systems: skin (lupus pernio, papular or plaque lesions, erythema nodosum); extrathoracic lymph nodes (enlarged nodes outside the thorax confirmed by imaging or biopsy); ocular (uveitis, conjunctival granulomas, optic nerve involvement); salivary glands (parotid or submandibular enlargement); hepatic (imaging abnormalities, laboratory changes, or histological confirmation); splenic (imaging abnormalities and/or histology confirmation); renal (granulomatous interstitial nephritis, nephrocalcinosis, or sarcoid-related hypercalcemia with renal dysfunction); neurologic (cranial neuropathies, central or peripheral nervous system involvement, or cerebrospinal fluid findings consistent with sarcoidosis); and cardiac (arrhythmias or conduction abnormalities, and/or diagnostic evidence on magnetic resonance imaging, positron emission tomography, or biopsy).
- Multiorgan disease was defined as involvement of three or more organs at diagnosis. Alternative thresholds included two or more and four or more organs involved.

Analytical parameters included hemoglobin, total leukocyte count, neutrophils, lymphocytes, monocytes, eosinophils, platelets, urea, creatinine, sodium, potassium, alanine aminotransferase (ALT), aspartate aminotransferase (AST), bilirubin, erythrocyte sedimentation rate (ESR), and C-reactive protein (CRP).

From the diagnostic CBC, absolute neutrophil, lymphocyte, monocyte, and platelet counts were used to compute the following indices:

- NLR = absolute neutrophil count/absolute lymphocyte count
- PLR = absolute platelet count/absolute lymphocyte count
- LMR = absolute lymphocyte count/absolute monocyte count

All hematological parameters were measured using automated hematology analyzers based on impedance and optical scatter cytometry, incorporating three-part and five-part differential leukocyte analysis for leukocyte, erythrocyte, and platelet quantification. Biochemical variables (urea, creatinine, electrolytes, liver enzymes, bilirubin, ESR, and CRP) were obtained through automated clinical chemistry analyzers using standardized photometric, ion-selective electrode, or immunoturbidimetric methods, according to manufacturer specifications.

All instruments operated under daily internal quality controls and periodic external proficiency testing, ensuring analytical stability and consistency throughout the study period.

### Statistical analysis

Continuous variables were summarized as medians with interquartile ranges (IQR) given non-normality, assessed with the Shapiro–Wilk test and visual inspection of histograms and Q-Q plots. Comparisons between groups were made using the Mann–Whitney U test. Categorical variables were presented as counts and percentages and compared using the chi-square test or Fisher’s exact test, as appropriate.

For each demographic (sex and ethnicity) and clinical characteristic (pulmonary involvement, extrapulmonary manifestations, and multiorgan disease), median values of the absolute hematologic counts (neutrophils, lymphocytes, monocytes, and platelets) were compared across the corresponding groups. Subsequently, the hematologic ratios derived from these counts (NLR, PLR, and LMR) were compared across the same categories.

The distribution of all continuous hematologic ratios was systematically assessed using descriptive statistics, graphical inspection (histograms and Q–Q plots), and skewness coefficients. When marked right-skewness (skewness coefficient >2) was identified, sensitivity analyses using log-transformed versions of the affected variables were pre-specified to evaluate robustness to distributional asymmetry. Primary analyses were conducted using the original scale of the hematologic ratios to preserve clinical interpretability.

For each organ-specific manifestation (present vs. absent), separate multivariable binary logistic regression models adjusted for age and sex were fitted. To avoid multicollinearity among the hematologic ratios, NLR, PLR, and LMR were analyzed in separate models, each entered individually as the hematologic predictor. Odds ratios (ORs) with 95% confidence intervals (CIs) were reported.

To assess factors associated with multiorgan disease (defined as involvement of three or more organs), univariate analyses were performed for all hematologic and biochemical variables (hemoglobin, leukocytes, neutrophils, lymphocytes, platelets, monocytes, eosinophils, urea, creatinine, sodium, potassium, ALT, AST, bilirubin, ESR and CRP) as well as NLR, PLR, LMR, age, and sex. Variables with *p* < 0.20 in univariate analyses were entered into multivariable logistic regression models. Because of multicollinearity with NLR, PLR, and LMR, absolute neutrophil, lymphocyte, monocyte, and platelet counts were excluded from multivariable models. As in the organ-specific analyses, NLR, PLR, and LMR were examined in separate models.

To further examine the relationship between hematologic indices and the extent of organ involvement, we constructed an ordinal logistic regression model (proportional odds model) using the number of organs involved as an ordinal outcome. The model included age, sex, and the laboratory variables that showed a *p* < 0.20 in univariate analyses. NLR, PLR, and LMR were entered in separate models to avoid multicollinearity. The proportional odds assumption was assessed using the test of parallel lines. Model coefficients were reported as log-odds with corresponding *p*-values, and exponentiated coefficients were interpreted as ORs representing the likelihood of belonging to a higher category of organ involvement.

Model diagnostics were systematically performed for all logistic and ordinal regression models. Multicollinearity was assessed using variance inflation factors (VIF), with all values <2 indicating minimal variance inflation. Events-per-variable (EPV) ratios were calculated to evaluate model stability based on the number of outcome events relative to the number of predictors included; EPV values <10 were considered indicative of potentially unstable estimates and increased risk of small-sample bias. Potential complete or quasi-complete separation was assessed by examining model convergence, finiteness of maximum likelihood estimates, and predicted probabilities; no evidence of separation was observed. For models with limited event counts, sensitivity analyses using penalized logistic regression (Firth correction; penalized maximum likelihood with Jeffreys prior) were conducted to mitigate potential small-sample bias. Penalized estimates were compared with maximum likelihood estimates to assess robustness of effect sizes.

Given the exploratory nature of the study and the performance of multiple hypothesis tests across organ involvement outcomes and hematologic indices, *p*-values from all primary multivariable logistic and ordinal regression models were adjusted for multiple comparisons using the Benjamini-Hochberg false discovery rate (FDR) procedure, controlling the expected proportion of false positives at 5%. Adjusted *p*-values are referred to as q-values. Both unadjusted p-values and q-values are reported for transparency. Statistical significance for the primary multivariable analyses was defined as a two-sided q-value < 0.05 (FDR-adjusted), whereas a nominal (unadjusted) *p* < 0.05 was considered statistically significant for descriptive and univariate comparisons.

Receiver operating characteristic (ROC) analyses were first performed for those organ-specific manifestations that showed independent associations in multivariable models, in order to evaluate the discriminative ability of NLR, PLR, and LMR for each outcome. ROC curves were generated and the area under the curve (AUC) was calculated for the relevant hematologic index in each organ-specific analysis. Optimal cut-off points were derived using the Youden index.

Subsequently, discriminative performance for multiorgan involvement was assessed. The primary definition of multiorgan disease was ≥3 organs involved. In addition, alternative definitions were evaluated using thresholds of ≥2 organs and ≥4 organs. For each of these three thresholds, ROC curves were generated for NLR, PLR, and LMR, and AUC values were calculated. Cut-off values were determined using the Youden index, the Euclidean minimum distance to the top-left corner of the ROC space, and high-sensitivity thresholds when clinically relevant.

All tests were two-sided, with *p* < 0.05 considered statistically significant. Analyses were conducted using IBM SPSS Statistics, version 29.0 (IBM Corp., Armonk, NY, USA), and Stata/MP, version 18 (StataCorp, College Station, TX, USA).

## Results

### Demographic and clinical baseline characteristics of the cohort

A total of 229 patients met inclusion criteria. The mean age at diagnosis was 51.34 ± 15.61 years, and 57.64% were women. Most patients were White (88.65%). Pulmonary involvement was present in 92.14% of cases, predominantly Scadding stage II (54.60%).

Extrapulmonary manifestations were frequent, most commonly cutaneous (36.68%) and extrathoracic lymph node involvement (18.78%). Less frequent manifestations included ocular, hepatic, joint, splenic, neurologic, salivary gland, renal, and cardiac involvement (see Table 1 for full distribution).

**Table 1:** Comparison of median values of CBCs counts stratified by demographic and clinical characteristics.

Variables (*n* %)		Neutrophils	Lymphocytes	Monocytes	Platelets	p-value
Sex						
Male (97, 42.46%)		4.30 (3.21–6.14)	1.67 (1.23–2.37)	0.62 (0.51–0.76)	285.00 (266.00–296.00)	Neutrophils:0.271 Lymphocytes: 0.244 Monocytes: 0.406 Platelets: 0.435
Female (132, 57.64%)		5.00 (3.61–6.68)	1.91 (1.37–2.59)	0.60 (0.46–0.76)	285.00 (266.00–296.00)	
Ethnicity						
White (203, 88.65%)		4.90 (2.75–5.93)	1.60 (1.06–2.23)	0.61 (0.42–0.77)	283.00 (275.50–308.50)	Neutrophils: 0.391 Lymphocytes: 0.301 Monocytes:0.878 Platelets: 0.452
Non-White (26, 11.35%)		4.69 (3.50–6.67)	1.80 (1.30–2.55)	0.60 (0.48–0.76)	285.00 (266.00–298.00)	
Pulmonary involvement. Scadding scale						
Stage 0 (20, 8.73%)	No	4.66 (3.50–6.54)	1.78 (1.34–2.43)	0.61 (0.49–0.76)	285.00 (268.00–298.75)	Neutrophils: 0.896 Lymphocytes: 0.818 **Monocytes: 0.017** Platelets: 0.574
	Yes	5.19 (3.04–7.13)	1.96 (1.19–2.75)	0.48 (0.35–0.64)	279.00 (269.50–299.75)	
Stage I (53, 23.14%)	No	4.72 (3.17–6.42)	1.77 (1.24–2.40)	0.61 (0.47–0.76)	285.00 (2.69–300.00)	Neutrophils: 0.424 Lymphocytes: 0.295 Monocytes: 0.686 Platelets: 0.287
	Yes	4.78 (3.74–6.84)	1.84 (1.44–2.62)	0.60 (0.51–0.75)	281.00 (263.60–298.00)	
Stage II (125, 54.59%)	No	4.92 (3.58–6.84)	1.84 (1.35–2.61)	0.60 (0.47–0.74)	279.00 (266.00–297.75)	Neutrophils: 0.405 Lymphocytes: 0.311 Monocytes: 0.611 Platelets: 0.090
	Yes	4.66 (3.09–6.26)	1.78 (1.25–2.40)	0.61 (0.48–0.77)	288.00 (269.00–302.00)	
Stage III (20, 8.73%)	No	4.79 (3.47–6.67)	1.81 (1.29–2.49)	0.60 (0.47–0.76)	285.00 (249.00–299.75)	Neutrophils: 0.724 Lymphocytes: 0.618 Monocytes: 0.674 Platelets: 0.365
	Yes	4.42 (3.29–6.41)	1.62 (1.26–2.34)	0.62 (0.48–0.71)	276.00 (263.25–294.25)	
Stage IV (11, 4.80%)	No	4.73 (3.29–6.62)	1.80 (1.28–2.45)	0.60 (0.47–0.76)	285.00 (268.00–298.50)	Neutrophils: 0.316 Lymphocytes: 0.517 Monocytes: 0.086 Platelets: 0.931
	Yes	5.44 (4.10–9.51)	1.84 (1.35–3.57)	0.73 (0.53–0.94)	279.00 (276.00–309.00)	
Extrapulmonary involvement						
Skin (84, 36.68%)	No	5.08 (3.50–6.66)	1.80 (1.34–2.57)	0.59 (0.47–0.74)	285.00 (269.00–298.00)	Neutrophils: 0.267 Lymphocytes: 0.283 Monocytes: 0.228 Platelets: 0.691
	Yes	4.55 (3.18–6.21)	1.79 (1.19–2.37)	0.64 (0.49–0.82)	280.00 (264.50–300.50)	
Extrathoracic lymph nodes (43, 18.78%)	No	4.88 (3.56–6.46)	1.88 (1.35–2.56)	0.61 (0.47–0.76)	285.00 (268.00–299.00)	Neutrophils: 0.277 **Lymphocytes: 0.006** Monocytes: 0.850 Platelets: 0.868
	Yes	4.46 (2.85–6.86)	1.58 (1.04–2.04)	0.59 (0.49–0.76)	283.00 (266.00–298.00)	
Splenic (15, 6.55%)	No	4.71 (3.45–6.42)	1.81 (1.29–2.48)	0.60 (0.47–0.75)	285.00 (268.00–299.25)	Neutrophils: 0.364 Lymphocytes: 0.600 Monocytes: 0.185 Platelets: 0.264
	Yes	5.36 (3.29–8.47)	1.64 (1.35–2.16)	0.67 (0.56–0.87)	285.00 (254.00–293.0)	
Central nervous system (13, 5.68%)	No	4.75 (3.50–6.63)	1.81 (1.35–2.50)	0.61 (0.48–0.76)	284.50 (267.00–298.00)	Neutrophils: 0.368 Lymphocytes: 0.080 Monocytes: 0.425 Platelets: 0.249
	Yes	4.08 (2.68–6.79)	1.18 (0.90–2.23)	0.52 (0.40–0.81)	287.00 (279.50–302.00)	
Hepatic (17, 7.42%)	No	4.75 (3.48–6.41)	1.78 (1.28–2.43)	0.61 (0.48–0.76)	285.00 (269.00–300.75)	Neutrophils: 0.802 Lymphocytes: 0.594 Monocytes: 0.365 Platelets: 0.068
	Yes	4.08 (3.07–9.14)	1.50 (1.06–3.40)	0.56 (0.45–0.69)	266.00 (262.50–293.00)	
Salivary glands (12, 5.24%)	No	4.62 (3.35–6.36)	1.78 (1.28–2.43)	0.61 (0.47–0.76)	285.00 (268.00–299.00)	Neutrophils: 0.077 Lymphocytes: 0.528 Monocytes: 0.704 Platelets: 0.489
	Yes	6.84 (3.76–9.41)	1.93 (1.36–3.35)	0.57 (0.48–0.93)	281.00 (264.00–293.25)	
Joint (17, 7.42%)	No	4.10 (3.50–6.67)	1.82 (1.34–2.53)	0.60 (0.47–0.75)	284.50 (286.00–298.00)	Neutrophils: 0.102 **Lymphocytes: 0.047** Monocytes: 0.454 Platelets: 0.641
	Yes	4.83 (2.73–5.43)	1.49 (1.11–1.85)	0.61 (0.49–0.89)	291.00 (255.00–303.00)	
Renal (4, 1.75%)	No	4.62 (3.34–6.40)	1.78 (1.28–2.41)	0.60 (0.47–0.75)	284.00 (267.50–298.50)	**Neutrophils: 0.013 Lymphocytes: 0.029** Monocytes: 0.675 Platelets: 0.159
	Yes	8.40 (6.49–12.75)	3.20 (2.16–3.82)	0.65 (0.49–0.84)	294.50 (286.50–313.75)	
Ocular (20, 8.73%)	No	4.80 (3.51–6.71)	1.84 (1.35–2.57)	0.61 (0.48–0.76)	285.00 (267.00–299.00)	Neutrophils: 0.032 **Lymphocytes: 0.010** Monocytes: 0.478 Platelets: 0.838
	Yes	4.04 (2.85–5.11)	1.41 (1.09–1.93)	0.57 (0.38–0.74)	285.00 (275.00–294.75)	
Cardiac (2, 0.87%)	No	4.73 (3.47–6.58)	1.80 (1.29–2.43)	0.60 (0.47–0.76)	285.00 (268.00–299.00)	Neutrophils: 0.700 Lymphocytes: 0.789 Monocytes: 0.238 Platelets: 0.427
	Yes	4.57 (1.99–7.14)	1.87 (0.93–2.80)	0.77 (0.69.0.85)	277.50 (264.00–291.00)	
Systemic involvement						
Multiorgan (55, 24.02%)	No	4.84 (3.52–6.36)	1.88 (1.35–2.55)	0.61 (0.48–0.76)	285.00 (269.00–300.25)	Neutrophils: 0.586 Lymphocytes: 0.052 Monocytes: 0.929 Platelets: 0.484
	Yes	4.60 (2.88–7.03)	1.60 (1.14–2.16)	0.59 (0.47–0.76)	284.00 (263.00–296.50)	

Data are presented as medians (IQR) and, when applicable, as minimum-maximum values. Comparisons between groups were performed using the Mann–Whitney U test. *p* < 0.05 was considered statistically significant. Organ involvement was determined using the WASOG 2014 tool. Bold values indicate statistically significant results (*p* < 0.05).

Overall, 61.14% of patients had involvement of at least two organs, 24.02% had three or more organs, and 8.73% had four or more organs affected.

### Hematologic counts and ratios according to demographic and clinical characteristics

In the overall cohort, the median cell counts were: neutrophils 4.73 × 10^9^/L [IQR 3.47–6.58], lymphocytes 1.80 × 10^9^/L [IQR 1.29–2.43], monocytes 0.60 × 10^9^/L [IQR 0.48–0.76], and platelets 285.00 × 10^9^/L [IQR 268.00–298.00]. Table 1 shows the median values (with IQR) of absolute hematologic counts, neutrophils, lymphocytes, monocytes, and platelets, stratified by demographic variables, pulmonary radiological stages, and extrapulmonary and multiorgan involvement.

When stratifying hematologic counts by demographic variables, values were largely similar, with no relevant differences observed between sex and ethnic groups.

Pulmonary characteristics were not associated with significant differences across Scadding stages. Most hematologic parameters showed no significant differences; however, monocyte counts were lower in patients with thoracic involvement.

Most extrapulmonary sites showed stable values across hematologic parameters. Exceptions included lower lymphocyte counts in extrathoracic lymph node involvement, joint involvement, ocular disease, and renal involvement. By contrast, renal involvement was the only manifestation characterized by higher neutrophil counts.

According to hematologic ratios, the overall median NLR was 2.57 [IQR 2.49–2.64], the PLR 158.97 [IQR 113.50–229.10], and the LMR 3.17 [IQR 2.07–4.44]. NLR, PLR, and LMR demonstrated positive skewness (NLR: mean 2.64; median 2.57; skewness coefficient = 2.59; PLR: mean 172.84; median 158.97; skewness coefficient = 1.18; LMR: mean 3.63; median 3.17; skewness coefficient = 1.66).

Table 2 shows the median values (IQR) of these ratios stratified by demographic variables, pulmonary radiological stages, and extrapulmonary organ involvement. Hematologic ratios remained consistent across sex and ethnicities.

**Table 2:** Comparison of median values of NLR, PLR, and LMR stratified by demographic and clinical covariates.

Variables (*n,* %)		NLR	PLR	LMR	*p*-value
Sex					
Male (97, 42.36%)		2.55 (2.50–2.64)	150.10 (112.39–203.14)	3.21 (2.17–4.66)	NLR: 0.448 PLR: 0.182 LMR: 0.168
Female (132, 57.64%)		2.57 (2.51–2.66)	169.58 (121.18–235.35)	2.84 (2.07–3.90)	
Ethnicity					
White (203, 88.65%)		2.57 (2.49–2.64)	154.67 (110.72–217.74)	3.17 (2.13–4.47)	NLR: 0.763 PLR: 0.268 LMR: 0.624
Non-White (26, 11.35%)		2.53 (2.47–2.71)	176.53 (127.83–259.83)	3.02 (2.00–4.27)	
Pulmonary involvement Scadding scale					
Stage 0 (20, 8.73%)	No	2.57 (2.50–2.66)	162.98 (114.97–228.24)	2.88 (2.07–4.22)	**NLR: 0.015** PLR: 0.682 **LMR: 0.038**
	Yes	2.54 (2.44–2.56)	142.27 (91.72–237.54)	4.47 (3.17–5.85)	
Stage I (53, 23.14%)	No	2.57 (2.50–2.66)	162.48 (115.13–237.37)	3.08 (2.08–4.64)	NLR: 0.822 PLR: 0.295 LMR: 0.927
	Yes	2.55 (2.49–2.62)	148.57 (108.93–194.81)	3.12 (2.07–4.08)	
Stage II (125, 54.59%)	No	2.56 (2.49–2.63)	150.89 (108.77–203.88)	3.31 (2.14–4.50)	NLR: 0.717 PLR: 0.271 LMR: 0.423
	Yes	2.57 (2.50–2.66)	162.98 (121.06–242.87)	2.98 (2.04–4.42)	
Stage III (20, 8.73%)	No	2.55 (2.49–2.64)	159.69 (114.80–229.40)	3.14 (2.07–4.47)	NLR: 0.197 PLR: 0.656 LMR: 0.443
	Yes	2.63 (2.59–2.70)	187.52 (103.53–227.55)	2.32 (2.18–6.03)	
Stage IV (11, 4.80%)	No	2.56 (2.50–2.64)	161.81 (113.34–229.97)	3.08 (2.12–4.47)	NLR: 0.265 PLR: 0.585 LMR: 0.967
	Yes	2.63 (2.47–3.43)	156.22 (103.92–206.81)	2.58 (1.63–5.38)	
Extrapulmonary involvement					
Skin (84, 36.68%)	No	2.57 (250–2.65)	160.42 (108.99217.84)	3.33 (2.14–4.65)	NLR: 0.815 PLR: 0.312 LMR: 0.096
	Yes	2.55 (2.48–2.65)	163.11 (124.81–242.87)	2.83 (1.92–3.96)	
Extrathoracic lymph nodes (43, 18.78%)	No	2.55 (2.49–2.63)	151.08 (110.42–214.84)	3.21 (2.14–4.61)	**NLR: 0.002** **PLR: 0.006** LMR: 0.061
	Yes	2.62 (2.51–3.46)	187.04 (142.00–274.42)	2.52 (1.93–3.72)	
Splenic (15, 6.55%)	No	2.55 (2.49–2.63)	161.70 (112.94–229.54)	3.17 (2.08–4.56)	**NLR:<0.001** PLR: 0.881 LMR: 0.151
	Yes	3.32 (2.71–3.84)	173.65 (118.66–233.66)	2.32 (1.91–3.37)	
Central nervous system (13, 5.68%)	No	2.56 (2.50–2.64)	161.06 (113.07–217.82)	3.08 (2.08–4.47)	NLR: 0.253 PLR: 0.053 LMR: 0.443
	Yes	2.64 (2.53–3.62)	204.46 (136.49–335.58)	3.03 (1.37–4.57)	
Hepatic (17, 7.42%)	No	2.55 (2.50–2.64)	160.42 (114.97–222.67)	3.06 (2.08–4.42)	**NLR: 0.013** PLR: 0.834 LMR: 0.843
	Yes	2.85 (2.53–3.48)	179.96 (90.28–269.70)	3.23 (1.81–5.85)	
Salivary glands (12, 5.24%)	No	2.56 (2.49–2.64)	161.95 (115.13–231.70)	3.11 (2.07–4.46)	**NLR: 0.029** PLR: 0.468 LMR:0.907
	Yes	2.82 (2.52–3.43)	123.33 (81.02–190.66)	2.83 (2.09–7.54)	
Joint (17, 7.42%)	No	2.57 (2.50–2.64)	158.61 (112.77–220.26)	2.15 (1.55–2.50)	NLR: 0.927 **PLR: 0.048** **LMR:0.018**
	Yes	2.52 (2.36–2.87)	200.31 (134.81–291.58)	3.19 (2.14–4.48)	
Renal (4, 1.75%)	No	2.56 (2.50–2.64)	162.48 (115.57–230.82)	3.05 (2.07–4.44)	NLR: 0.166 **PLR: 0.049** LMR: 0.124
	Yes	2.99 (2.51–3.51)	95.55 (74.96–147.35)	4.94 (3.09–6.51)	
Ocular (20, 8.73%)	No	2.57 (2.50–2.64)	158.26 (110.52–222.67)	3.12 (2.07–4.47)	NLR: 0.899 **PLR: 0.008** LMR: 0.175
	Yes	2.52 (2.49–3.38)	191.79 (150.14–245.70)	2.82 (2.14–3.46)	
Cardiac (2, 0.87%)	No	2.57 (2.50–2.65)	161.81 (114.35–229.11)	3.08 (2.08–4.47)	NLR: 0.206 PLR: 0.805 LMR: 0.521
	Yes	2.35 (2.14–2.55)	203.06 (94.40–311.79)	2.57 (1.08–4.05)	
Systemic involvement					
Multiorgan (55, 24.02%)	No	2.55 (2.50–2.62)	152.03 (110.42–214.14)	3.29 (2.19–4.47)	**NLR: 0.049** PLR: 0.065 LMR: 0.107
	Yes	2.63 (2.46–3.36)	177.21 (128.20–251.38)	2.66 (1.90–3.79)	

Data are presented as medians (IQR) and, when applicable, as minimum-maximum values. Comparisons between groups were performed using the Mann–Whitney U test. *p* < 0.05 was considered statistically significant. Organ involvement was determined using the WASOG 2014 tool. Bold values indicate statistically significant results (*p* < 0.05).

No significant differences were observed across pulmonary involvement. However, patients without radiographic pulmonary disease (Scadding stage 0) had a lower NLR and a higher LMR whereas PLR did not vary significantly across this stage.

Distinct patterns emerged across extrapulmonary sites. Extrathoracic lymph node disease was associated with concurrent elevations in NLR and PLR. Higher NLR values were also observed in splenic, hepatic, and salivary gland involvement. PLR was increased in ocular disease and reduced in renal involvement, while joint manifestations were associated with modestly higher LMR values. Detailed medians and statistical comparisons are presented in Table 2.

### Diagnostic and discriminatory value of NLR, PLR, and LMR in organ specific manifestations

For each organ-specific manifestation, separate multivariable binary logistic regression models including age, sex, and one hematologic ratio (NLR, PLR, or LMR) were fitted. After adjustment for multiple comparisons using the Benjamini-Hochberg FDR procedure, significant independent associations were observed only for NLR and PLR with extrathoracic lymph node involvement (NLR: OR = 13.42, 95% CI 1.91–94.32, *p* = 0.001, q = 0.005; PLR: OR = 1.01, 95% CI 1.00–1.01, *p* = 0.008, q = 0.040; 43 events; EPV = 14.33) and for NLR with splenic involvement (OR = 83.05, 95% CI 6.75–1021.09, *p* = 0.001, q = 0.005; 15 events; EPV = 5.00).

In sensitivity analyses, penalized logistic regression was applied to the low-event splenic involvement model. Under penalized maximum likelihood estimation, the magnitude of the association was reduced, as expected in low-event settings, but remained statistically significant and directionally consistent (OR = 23.72; 95% CI 7.53–74.70). Separately, given the right-skewed distribution observed for NLR, log-transformed NLR was evaluated to assess robustness to distributional asymmetry. In the splenic involvement model, log (NLR) remained significantly associated with splenic disease, corresponding to an OR of 2.74 per 10% increase in NLR (95% CI 1.92–3.91; *p* < 0.001). For extrathoracic lymph node involvement, log-transformed NLR was also significantly associated, corresponding to an OR of 2.17 per 10% increase in NLR (95% CI 1.60–2.94; *p* < 0.001). No other organ-specific manifestations showed independent associations after FDR correction.

Additionally, we found no association between Scadding stage and NLR, PLR, or LMR in multivariable analyses; likewise, across other extrapulmonary sites, no significant associations were observed for any hematologic index with cardiac, cutaneous, ocular, renal, hepatic, or neurologic involvement.

**Figure 1:**
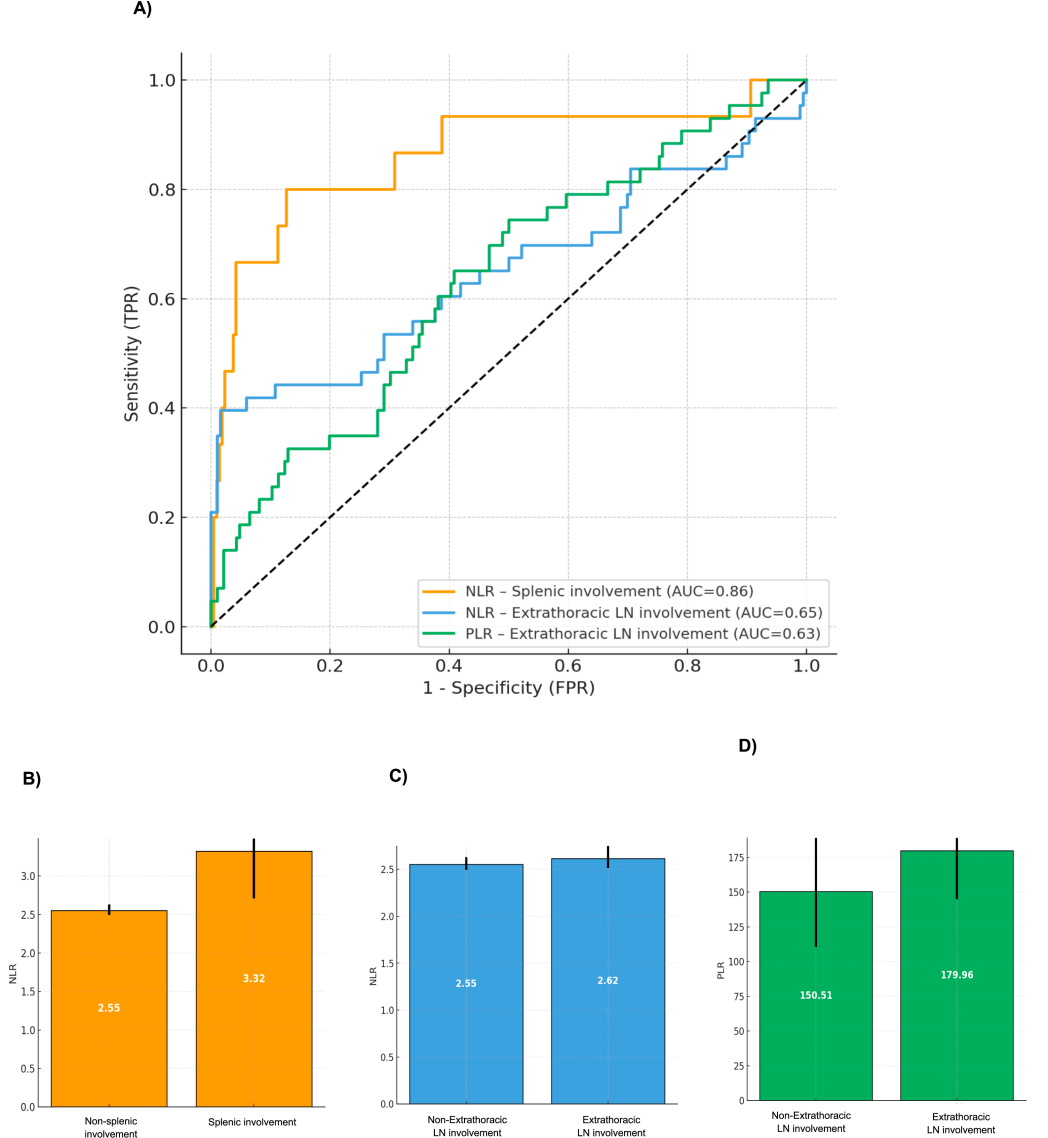
Assessment of hematologic ratios in organ-specific sarcoidosis involvement. **(A)** Combined ROC curves for hematologic ratios in organ specific sarcoidosis involvement; **(B)** median and IQR of NLR by splenic involvement; **(C)** median and IQR of NLR by extrathoracic LN involvement; **(D)** median and IQR of PLR by extrathoracic LN involvement. AUC, area under the ROC Curve; IQR, interquartile range; NLR, neutrophil-to-lymphocyte ratio; PLR, platelet-to-lymphocyte ratio; LN, lymph node.

**Figure 2:**
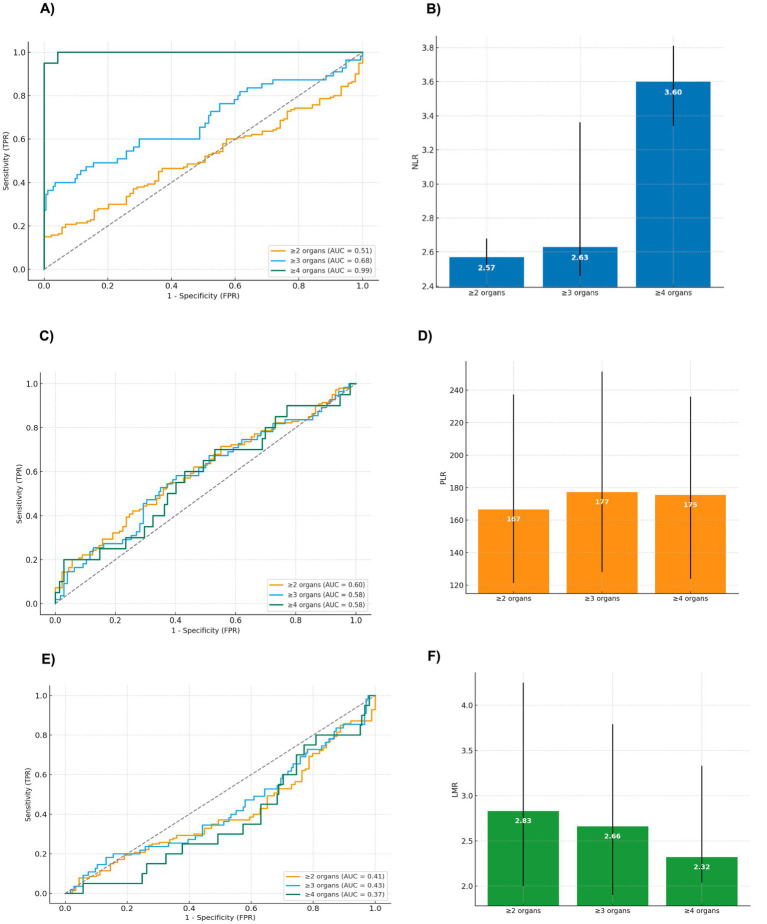
ROC curves of NLR, PLR, and LMR for multiorgan involvement defined as ≥2, ≥3, or ≥4 organs. **(A)** ROC curves of NLR according multiorgan involvement; **(B)** median and IQR of NLR by multiorgan involvement; **(C)** ROC curves of PLR according multiorgan involvement; **(D)** median and IQR of PLR by multiorgan involvement; **(E)** ROC curves of LMR according multiorgan involvement; **(F)** median and IQR of LMR by multiorgan involvement. AUC, area under the ROC curve; IQR, interquartile range; NLR, neutrophil-to-lymphocyte ratio; PLR, platelet-to-lymphocyte ratio.

Based on these results, ROC curve analyses were performed exclusively for the hematologic indices that showed statistically significant associations in the multivariable models (Figure 1). Specifically, splenic disease showed good discrimination with NLR (AUC = 0.86), with an optimal cut-off of NLR ≥ 2.70 providing 80.00% sensitivity and 87.40% specificity. For extrathoracic lymph node disease, NLR demonstrated moderate discrimination (AUC = 0.65) with an optimal cut-off of 2.86 (sensitivity 39.50%, specificity 98.40%). The ROC curve analysis evaluating PLR as a predictor of extrathoracic lymph node involvement yielded an AUC of 0.63, indicating modest discriminative ability. The optimal cut-off point, determined by the Youden index, was PLR > 150.6, providing 74.40% sensitivity and 50.00% specificity (Figure 2).

### Diagnostic and discriminatory value of NLR, PLR, and LMR in multiorgan sarcoidosis

When median values were measured for the different ratios and definitions of multiorgan involvement, we found that NLR increased with the number of organs affected (≥2: 2.57 [IQR 2.49–2.68]; ≥3: 2.63 [IQR 2.46–3.36]; ≥4: 3.60 [IQR 3.34–3.81]), PLR remained relatively stable (≥2: 166.59 [IQR 121.36–237.32]; ≥3: 177.21 [IQR 128.20–251.38]; ≥4: 175.43 [IQR 123.98–235.99]), and LMR decreased progressively (≥2: 2.83 [IQR 2.00–4.25]; ≥3: 2.66 [IQR 1.90–3.79]; ≥4: 2.32 [IQR 2.04–3.33]).

Differences in baseline laboratory parameters according to the presence or absence of multiorgan involvement are summarized in Table 3.

**Table 3:** Median values of analytics parameters in multiorgan and non-multiorgan involvement.

Variable	Multiorgan involvement *n* = 55 (median, IQR 25–75)	Non-multiorgan involvement *n* = 174 (median, IQR 25–75)	*p*-value
Hemoglobin (g/dl)	14.18 (13.38–15.02)	14.07 (13.41–14.74)	0.489
Leukocytes (×10^9^/L)	7.17 (4.92–9.75)	7.77 (5.81–9.78)	0.379
Neutrophils (×10^9^/L)	4.57 (2.92–6.87)	4.84 (3.54–6.32)	0.587
Lymphocytes (×10^9^/L)	1.60 (1.13–2.12)	1.88 (1.35–2.54)	0.052
Monocytes(×10^9^/L)	0.60 (0.48–0.76)	0.61 (0.48–0.76)	0.930
Eosinophils (×10^9^/L)	0.29 (0.23–0.32)	0.29 (0.26–0.32)	0.386
Platelets (×10^9^/L)	283.00 (264.5–296.0)	285.00 (269.0–299.8)	0.484
Urea (mg/dL)	30.50 (21.40–37.55)	32.55 (24.02–38.35)	0.654
Creatinine (mg/dL)	1.01 (0.90–1.12)	0.98 (0.86–1.10)	0.404
Sodium (mmol/L)	140.00 (139.0–141.0)	140.00 (139.0–141.8)	0.284
Potassium (mmol/L)	4.16 (3.92–4.36)	4.17 (3.98–4.37)	0.640
ALT (U/L)	26.60 (19.00–34.85)	24.90 (18.73–31.35)	0.248
AST (U/L)	25.20 (22.50–27.80)	24.45 (21.45–27.50)	0.401
Bilirubin (mg/dL)	0.77 (0.64–0.99)	0.80 (0.61–1.00)	0.988
ESR (mm/h)	21.00 (17.25–25.30)	21.55 (17.90–27.50)	0.429
CRP (mg/dL)	1.00 (0.95–1.06)	0.99 (0.93–1.05)	0.459
NLR	2.63 (2.46–3.36)	2.55 (2.50–2.62)	**0.049**
PLR	177.21 (128.20–251.38)	152.03 (110.42–214.14)	0.065
LMR	2.66 (1.90–3.79)	3.29 (2.19–4.47)	0.107

Data are expressed as median (interquartile range, IQR). Comparisons between groups were performed using the Mann–Whitney U test. *p* < 0.05 was considered statistically significant. ALT = alanine aminotransferase; AST = aspartate aminotransferase; ESR = erythrocyte sedimentation rate; CRP = C-reactive protein; NLR = neutrophil-to-lymphocyte ratio; PLR = platelet-to-lymphocyte ratio; LMR = lymphocyte-to-monocyte ratio. Bold values indicate statistically significant results (*p* < 0.05).

In the stratified univariate analyses, hematologic ratios varied according to multiorgan involvement (defined as the involvement of ≥3 organs), in which NLR was significantly elevated. No significant differences were detected for PLR or LMR.

A multivariable binary logistic regression model was constructed with multiorgan involvement (≥3 organs) as the dependent variable. After FDR adjustment for multiple testing, each 1-unit increase in NLR was associated with an OR of 24.60 (95% CI 5.90–102.00, *p* < 0.001, q = 0.005; 55 events; EPV = 18.33) for multiorgan involvement; PLR and LMR were not independently associated. Given the marked right-skewed distribution observed for NLR, sensitivity analyses using log-transformed NLR were performed to assess robustness to distributional asymmetry. In the multivariable binary logistic regression model for multiorgan involvement (≥3 organs), log-transformed NLR remained significantly associated with disease extent, with an OR of 2.13 per 10% increase in NLR (95% CI 1.56–2.91; *p* < 0.001), consistent with the primary analysis using untransformed NLR.

The correlation between CBC-derived ratios and the number of organs involved was assessed using Spearman’s rank correlation coefficient. NLR showed a correlation coefficient of *ρ* = 0.23 (*p* = 0.035), PLR a coefficient of ρ = 0.17 (*p* = 0.045), and LMR a coefficient of ρ = −0.16 (*p* = 0.059). An ordinal logistic regression model was then applied using the number of organs involved as the dependent variable. After FDR correction, NLR was associated with an OR of 53.10 (95% CI 19.00–147.60, *p* < 0.001, q = 0.005) and PLR with an OR of 1.01 (95% CI 1.00–1.01, *p* = 0.001, q = 0.005) for higher categories of organ involvement.

To evaluate discriminative performance, ROC analyses were performed for NLR, PLR, and LMR under three predefined thresholds (Figure 2).

For NLR, ROC analysis across the three multiorgan definitions yielded AUC values of 0.51 (≥2 organs), 0.69 (≥3 organs), and 0.99 (≥4 organs). Using the primary definition (≥3 organs), the Youden index cut-off was 2.74 (sensitivity 43.60%, specificity 97.10%). The Euclidean minimum-distance point was 2.63 (sensitivity 50.90%, specificity 77.00%), and a high-sensitivity threshold of 2.43 produced 80.00% sensitivity with 8.00% specificity. Under alternative definitions, the ≥2-organ threshold resulted in an AUC of 0.51, with a Youden cut-off of 2.59 (sensitivity 46.00%, specificity 62.20%) and a minimum-distance cut-off of 2.57 (sensitivity 48.90%, specificity 48.90%). For ≥4 organs (AUC 0.99), both the Youden and Euclidean minimum-distance cut-offs were 2.74, with sensitivities of 95.00 and 100.00%, and specificity of 95.70%.

For PLR, AUC values were 0.60, 0.58, and 0.58 for the ≥2-, ≥3-, and ≥4-organ thresholds, respectively. For ≥3 organs, the Youden index identified an optimal cut-off of 177 (sensitivity 48.0%, specificity 71.0%). The minimum-distance cut-off was 168 (sensitivity 52.0%, specificity 62.0%), and a high-sensitivity threshold near 142 yielded 78.0% sensitivity with 22.0% specificity. Furthermore, for ≥2 organs (AUC 0.60), the Youden cut-off was 167 (sensitivity 56.0%, specificity 63.0%) and the minimum-distance point was 160 (sensitivity 60.0%, specificity 55.0%) and for ≥4 organs (AUC 0.58), the Youden cut-off was 175 (sensitivity 62.0%, specificity 66.0%) and the minimum-distance cut-off was 170 (sensitivity 68.0%, specificity 59.0%).

For LMR, AUC values were 0.41, 0.43, and 0.37 for the ≥2-, ≥3-, and ≥4-organ definitions, respectively. For LMR, ROC analyses were performed after reversing the direction of the variable (1/LMR), given its inverse relationship with disease severity. AUC values were 0.60 for ≥2 organs, 0.58 for ≥3 organs, and 0.63 for ≥4 organs. For ≥3 organs, the Youden index identified an optimal cut-off corresponding to LMR ≤ 3.23, providing 65.5% sensitivity and 51.1% specificity. For ≥2 organs, the Youden cut-off corresponded to LMR ≤ 3.18 (sensitivity 60.0%, specificity 62.9%), and for ≥4 organs, the Youden cut-off was LMR ≤ 2.66, yielding 65.0% sensitivity and 63.2% specificity.

## Discussion

In this large, well-characterized retrospective cohort of sarcoidosis patients, we demonstrate that simple hematologic inflammatory ratios, particularly the NLR and, to a lesser extent, the PLR, provide meaningful information about organ-specific and systemic disease involvement at diagnosis. NLR was significantly elevated in patients with certain extrapulmonary manifestations, showing clear associations with splenic involvement, extrathoracic lymphadenopathy, and multiorgan disease. PLR also correlated with extrathoracic lymph node involvement, although its predictive value for multiorgan involvement was limited, while the LMR showed no meaningful association with organ-specific or systemic involvement.

Furthermore, we established data-driven cut-off values indicating that specific NLR thresholds identify both splenic involvement and extrathoracic lymphadenopathy. PLR thresholds identify extrathoracic lymphadenopathy. Importantly, an NLR cut-off value accurately identifies multiorgan disease, underscoring the diagnostic value of these inexpensive, widely available ratios.

In extrapulmonary disease, the clearest organ-specific associations were observed for extrathoracic lymph node and splenic involvement. Both NLR and PLR were independently associated with extrathoracic lymphadenopathy, whereas NLR alone remained strongly linked to splenic disease after multivariable adjustment. ROC analyses identified clinically actionable cut-offs: NLR ≥ 2.86 and PLR ≥ 150.6 for extrathoracic lymph node involvement, and NLR ≥ 2.70 for splenic sarcoidosis (80.00% sensitivity, 87.40% specificity). These thresholds fall within the range of previously reported NLR cut-offs (2.0–3.5) described in studies of active or progressive sarcoidosis (31–35), providing site-specific reference values for these extrapulmonary phenotypes. Collectively, our data suggest that NLR and PLR may refine the clinical characterization of sarcoidosis by offering phenotype-oriented thresholds, particularly for splenic and extrathoracic lymph node involvement, supporting existing evidence that systemic inflammatory activation is linked to more disseminated disease (33, 36). Prospective studies are now needed to validate these thresholds and determine their utility in diagnostic pathways and risk stratification.

By contrast, hematologic ratios showed limited ability to differentiate the extent of intrathoracic involvement. NLR, PLR, and LMR were largely stable across Scadding stages I-IV. This differs from some prior reports linking higher NLR to parenchymal lung involvement (25, 36). However, our study uniquely applies extensive multivariable analyses anchored in a detailed mapping of extrapulmonary, organ-specific involvement and evaluates baseline (treatment-naïve) values at diagnosis. Moreover, although the Scadding system separates lymph node enlargement from parenchymal involvement and fibrosis, it provides limited granularity on parenchymal subtypes and does not delineate underlying inflammatory activity patterns; integrating computed tomography (CT) and fluorodeoxyglucose positron emission tomography/CT-based phenotyping may pinpoint which parenchymal patterns and activity profiles confer excess risk, supporting earlier, stage-informed management. Taken together, our data highlight NLR’s role as an integrative marker of total inflammatory load, rather than a direct gauge of lung disease alone as measured by Scadding staging.

Beyond single-organ involvement, NLR showed a consistent association with multiorgan disease across all analytical approaches. In multivariable models, higher NLR values were linked to greater odds of involvement of ≥3 organs, and this association persisted when the number of affected organs was modeled as an ordinal variable. PLR also showed a significant relationship in the ordinal analysis. Importantly, ROC analyses confirmed that NLR provided superior discriminative performance compared with PLR, underscoring its potential clinical utility. With regard to practical interpretability, the evaluation of potential cut-off values suggested thresholds adaptable to different clinical priorities. For NLR, a value close to 2.4 favored sensitivity, whereas values between 2.6 and 2.7 provided a more balanced trade-off sensitivity and specificity for detecting multiorgan involvement, and PLR, ROC analysis showed that values near 142 favored sensitivity, while values between 168 and 177 offered a more optimal balance between sensitivity and specificity. Previous studies evaluating disease activity, treatment need, or radiological severity have reported NLR thresholds between approximately 2.3 to 3.2 (25, 32–35), whereas for PLR a threshold of 158 has been described (37). However, studies specifically assessing multiorgan involvement remain scarce. Our findings extend prior evidence by showing that NLR is associated with the extent of organ involvement after multivariable adjustment and that PLR may provide complementary information. These parameters could be further evaluated as accessible indicators to guide the assessment of systemic disease burden.

In line with the observations of our study, the clinical relevance of CBC-derived inflammatory indices extends beyond sarcoidosis. NLR, PLR, and to a lesser extent LMR have been consistently associated with disease activity and organ involvement across multiple systemic inflammatory conditions, including rheumatoid arthritis, familial Mediterranean fever, systemic sclerosis, systemic lupus erythematosus, and inflammatory bowel disease (38–47). These findings support the biological plausibility of our results and suggest that hematologic ratios may reflect systemic inflammatory activity and organ involvement across diverse inflammatory disorders.

This study has several limitations. Its retrospective, single-center design relies on routinely collected clinical data, which may not consistently capture mild or subclinical organ involvement. Moreover, certain extrapulmonary manifestations were represented by a limited number of cases, a feature inherent to the heterogeneous clinical spectrum of sarcoidosis, and this should be considered when interpreting organ-specific effect estimates, including the magnitude of some odds ratios. In this context, the larger odds ratios observed for NLR in selected models should be interpreted with caution and in light of the mathematical and biological properties of this index. NLR is a composite ratio derived from neutrophil and lymphocyte counts, and a one-unit increase typically reflects a meaningful shift in inflammatory balance rather than a marginal change in a single laboratory parameter.

Furthermore, the study population, predominantly composed of white individuals and derived from a tertiary referral setting, may not fully reflect the demographic or clinical spectrum seen in broader community populations, potentially affecting the generalizability of the findings. Additionally, the analyses were confined to baseline hematologic parameters, which restricts the ability to assess temporal trends or their relationship with therapeutic response and disease trajectory.

Despite these limitations, our design mirrors real-world diagnostic practice and provides one of the most detailed single-center assessments of hematologic ratios in sarcoidosis to date. The study highlights the practical value of NLR and, to a lesser extent, PLR as low-cost, point-of-care adjuncts for risk stratification. Both indices derive from a routine complete blood count, they can be calculated immediately without added cost or specialized assays, making them attractive across resource-limited and high-resource settings alike. These ratios should complement established diagnostic tools. Integrating NLR (and selectively PLR) into baseline assessment offers clinicians a simple, universally available marker that correlates with systemic disease extent and can flag patients who may warrant more extensive evaluation.

In addition to demonstrating independent associations with organ-specific and multiorgan involvement, our analyses provide exploratory ROC-derived cut-off values that may assist clinicians in identifying patients at higher risk of systemic disease burden. These thresholds offer reference points that can support initial clinical assessment and prioritization of further evaluation. Prospective multicenter studies with serial measurements and external validation remain essential before formal incorporation into prognostic algorithms and therapeutic decision-making. Nevertheless, current evidence supports the inclusion of these ratios as accessible adjuncts in the clinical toolkit for sarcoidosis care.

## Conclusion

In this large retrospective cohort, NLR was independently associated with multiorgan disease, splenic and extrathoracic lymph node involvement. PLR was independently associated with extrathoracic lymph node involvement. These findings underscore the value of NLR as a simple, low-cost marker that may help characterize the multisystem nature of sarcoidosis and complement standard clinical, laboratory, and imaging assessments in the initial evaluation of the disease.

## Data Availability

The original contributions presented in the study are included in the article/supplementary material, further inquiries can be directed to the corresponding author.
